# Impact of COVID-19 on UK stress echocardiography practice: insights from the EVAREST sites

**DOI:** 10.1530/ERP-20-0043

**Published:** 2021-03-19

**Authors:** Cameron Dockerill, William Woodward, Annabelle McCourt, Cristiana Monteiro, Elena Benedetto, Maria Paton, David Oxborough, Shaun Robinson, Keith Pearce, Mark J. Monaghan, Daniel X. Augustine, Paul Leeson

**Affiliations:** 11 Cardiovascular Clinical Research Facility, RDM Division of Cardiovascular Medicine, University of Oxford, Oxford, UK; 21 School of Medicine, University of Leeds, Leeds, UK; 31 Cardiovascular Physiology, School of Sport and Exercise Sciences, Liverpool, UK; 41 North West Anglia NHS Foundation Trust, Bretton Gate, Bretton, Peterborough, Cambridgeshire, UK; 51 Manchester University NHS Foundation Trust, Wythenshawe Hospital, Wythenshawe, Manchester, UK; 61 King’s College Hospital NHS Foundation Trust, Denmark Hill, London, UK; 71 Royal United Hospitals Bath NHS Foundation Trust, Bath, UK; 81 Department for Health, University of Bath, Bath, Somerset, UK

**Keywords:** stress echocardiography, COVID-19, coronary artery disease, ischaemic heart disease, survey, national health services

## Abstract

*Introduction* Healthcare delivery is being transformed by COVID-19 to reduce transmission risk but continued delivery of routine clinical tests is essential. Stress echocardiography is one of the most widely used cardiac tests in the NHS. We assessed the impact of the first (W_1_) and second (W_2_) waves of the pandemic on the ability to deliver stress echocardiography.

*Methods* Clinical echocardiography teams in 31 NHS hospitals participating in the EVAREST study were asked to complete a survey on the structure and delivery of stress echocardiography as well as its impact on patients and staff in July and November 2020. Results were compared to stress echocardiography activity in the same centre during January 2020.

*Results* 24 completed the survey in July, and 19 NHS hospitals completed the survey in November. A 55% reduction in the number of studies performed was reported in W_1_, recovering to exceed pre-COVID rates in W_2_. The major change was in the mode of stress delivery. 70% of sites stopped their exercise stress service in W_1_, compared to 19% in W_2_. In those still using exercise during W_1_, 50% were wearing FFP3/N95 masks, falling to 38% in W_2_. There was also significant variability in patient screening practices with 7 different pre-screening questionnaires used in W_1_ and 6 in W_2_.

*Conclusion* Stress echocardiography delivery restarted effectively after COVID-19 with adaptations to reduce transmission that means activity has been able to continue, and exceed, pre-COVID-19 levels during the second wave. Further standardization of protocols for patient screening and PPE may help further improve consistency of practice within the United Kingdom.

*Conclusion* Stress echocardiography delivery restarted effectively after COVID-19 with adaptations to reduce transmission that means activity has been able to continue, and exceed, pre-COVID-19 levels during the second wave. Further standardization of protocols for patient screening and PPE may help further improve consistency of practice within the United Kingdom.

https://erp.bioscientifica.com
https://doi.org/10.1530/ERP-20-0043

## Introduction

The coronavirus (COVID-19) pandemic is placing unprecedented strain on healthcare services across the world (1), with the UK's National Health Service (NHS) experiencing greatest challenge in 70 years of its existence (2). The disease has spread rapidly across the United Kingdom (3) with now over 125,000 COVID-19-related deaths recorded in the country (3). The NHS activity restarted (4) following the first wave of COVID-19 infections but with new regulations to minimize patient's contact with the healthcare professionals and to reduce risk of transmission via aerosol generating procedures (5, 6, 7, 8). Stress echocardiography is one of the most widely used tests to assess cardiac function and to determine whether a patient has evidence of coronary ischaemia (9, 10, 11). Therefore, continued delivery of stress echocardiography is essential to provide effective healthcare within the NHS. We studied whether COVID-19 and its associated healthcare regulations had impacted the ability to deliver stress echocardiography in the NHS.

## Methods

The 'Impact of COVID-19 on UK Stress Echocardiography Services' survey was sent to the research teams from the 31 NHS hospitals participating in the EVAREST study (ClinicalTrials.gov ID: NCT03674255) in both July and November 2020. The EVAREST study is a UK-wide prospective stress echocardiography study that aims to evaluate real world performance, accuracy and cost of stress echocardiography and has been running since 2011. The existing network of the UK hospitals, set up as a part of the EVAREST study, provided the infrastructure to distribute the survey and collect results from hospitals across the UK. Survey results could be compared to historical data from the same centres based on the data collected by the EVAREST study as well as comparing the results between the two waves of COVID-19. The survey was developed as a consensus document in collaboration with the British Society of Echocardiography (BSE) and contained questions focussing on the impact of COVID-19 on stress echocardiography practice, patients and the NHS staff (Supplementary data, see section on supplementary materials given at the end of this article). The survey was deployed electronically to 31 NHS sites via Google Forms (Google LLC., Mountain View, California, United States). Responses from the sites were collated after 10 days.

### Statistical analysis

Hospital characteristics and reported data from the NHS sites were reported using standard approaches.

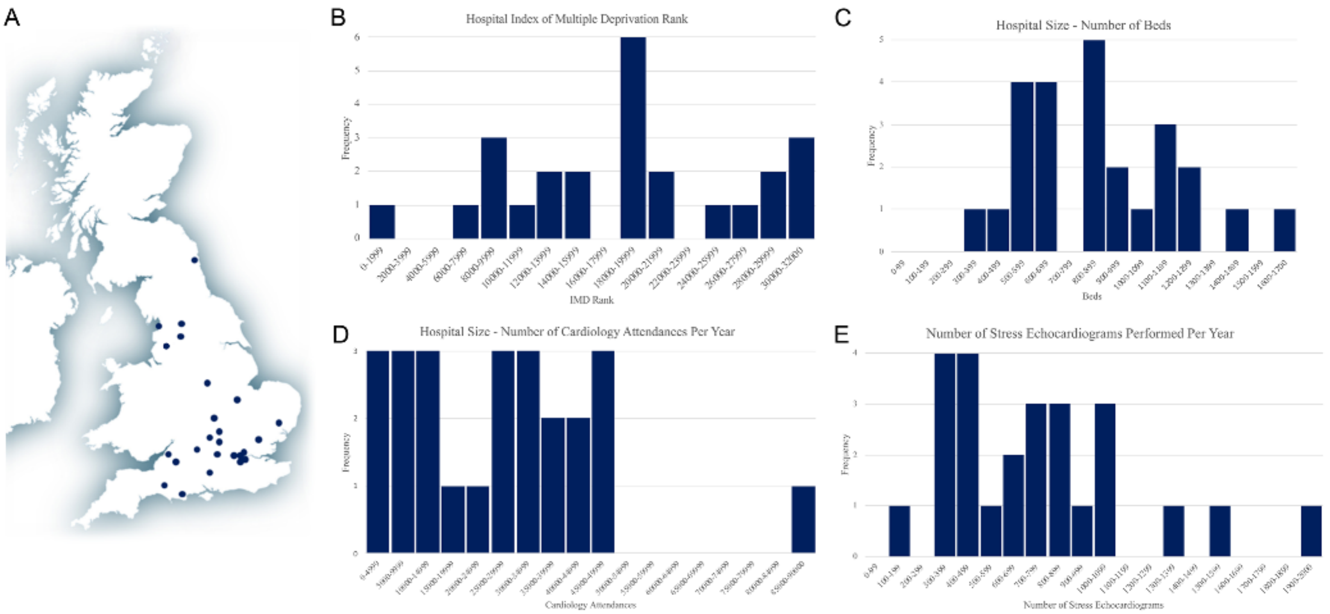

**Figure 1**
(A) Location of the 26 NHS hospital sites surveyed. (B) Range of IMD ranks for each hospital, demonstrating the wide range of socio-economic backgrounds represented (the lower the ranking, the greater the level of socio-economic deprivation). (C) Hospital size as measured by number of beds. (D) Hospital cardiology department size, as measured by number of cardiology attendances per year. (E) Volume of stress echocardiograms performed at each hospital per year. Two of the 24 hospitals who responded to the survey (Broomfield Hospital and Basildon Hospital) are grouped under the same trust name, therefore each graph represents data from 25 NHS trusts.

https://erp.bioscientifica.com
https://doi.org/10.1530/ERP-20-0043
© 2020 The authors
Published by Bioscientifica Ltd

To calculate the significance level of the difference in estimated numbers of stress echocardiography studies performed before and during the two waves of the pandemic, a paired two-tailed distribution *t*-test was used with a significance level of $P < 0.05$ (Microsoft Excel Version 16.39, Microsoft Corporation).

## Results

### Participating sites

A total of 24 NHS hospitals (77%) responded to the survey in July, immediately after the peak of the first wave ($W_1$) and 19 hospitals (61%) in November in the middle of the second wave ($W_2$). Seventeen hospitals responded to both the surveys. Data on the geographical spread, index of multiple deprivation, the number of hospital beds, the number of cardiology attendances and self-reported numbers of stress echocardiograms performed per annum at each site are presented in Fig. 1.

### Stress echo practice

Figure 2A illustrates that stress echocardiography was being performed at 21 sites (87.5%) with three having stopped their service entirely in $W_1$. During $W_2$, stress echocardiography was being performed at all 19 sites (100%), with eight sites (42%) reporting that their stress echocardiography service was now unaffected by COVID-19. The number of sessions being performed at sites was reduced during $W_1$ (range 1–11 sessions per week) compared to pre-COVID-19 (range 1–20), recovering back to a range of 1–20 sessions per week reported by sites during $W_2$. Figure 2D shows a reduction in the number of patients seen in each stress echocardiography session,

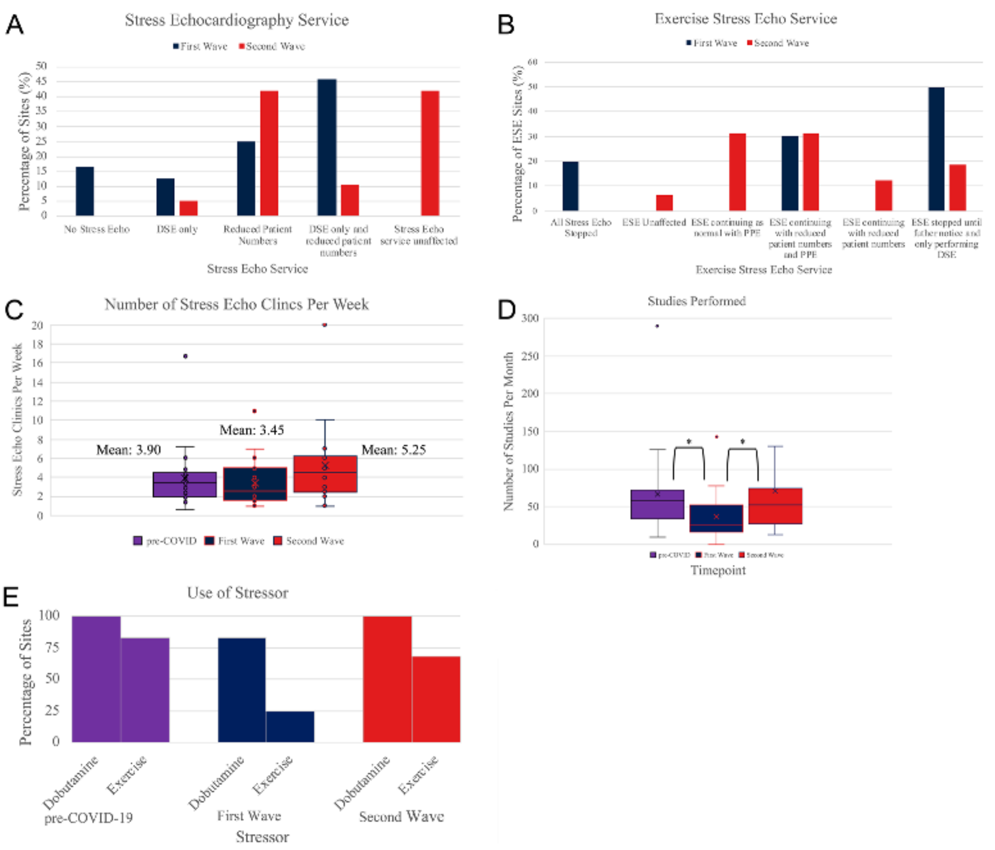

**Figure 2**

(A) Reported general stress echocardiography service at sites during the first wave ($n = 24$) and second wave ($n = 19$). (B) Reported exercise stress echocardiography service. Percentage reported as proportion of sites who performed ESE before COVID-19 ($n = 20$ $W_1$, $n = 16$ $W_2$). (C) The number of stress echocardiography sessions performed at the sites pre-COVID ($n = 25$), during the first wave ($n = 20$) and second wave ($n = 18$ – one site did not report). The number of sessions per week, pre-COVID, has been estimated using the reported annual number of studies, assuming four patients per session. (D) The reported number of patients seen in each stress echocardiography session, as reported in a sub-set of sites ($n = 7$). (E) The number of sites performing dobutamine and exercise stress echocardiography before COVID-19 ($n = 24$) and during the first ($n = 24$) and second ($n = 19$) waves.

https://erp.bioscientifica.com
https://doi.org/10.1530/ERP-20-0043
© 2020 The authors
Published by Bioscientifica Ltd

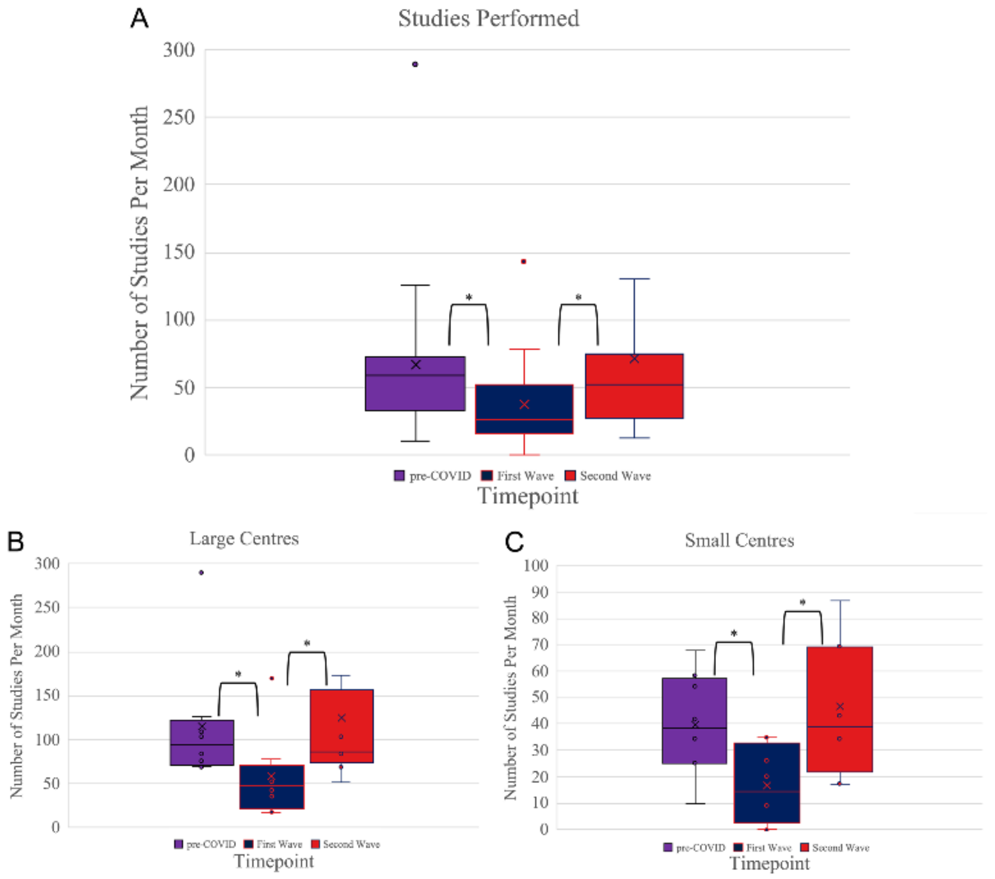

**Figure 3**

(A) The estimated number of stress echocardiography studies performed at each hospital site pre-COVID-19 (*n* = 16), during the first (*n* = 16) and second (*n* =16) waves. Pre-COVID-19 numbers are self-reported data previously collected as a part of the EVAREST study. Current COVID-19 numbers are an estimation based on the reported number of stress echocardiography sessions being performed weekly at each site, assuming four patients per session pre-COVID and three patients per session in $W_1$ and $W_2$, as reported in Fig. 2C. *n* number reduced as only sites who responded to both surveys ($W_1$ and $W_2$) and indicated number of sessions per week can be compared. (B) The estimated number of stress echocardiography studies performed at high volume centres (>800 stress echo studies per annum, *n* = 8) pre-COVID, during $W_1$ and during $W_2$. (C) The estimated number of stress echocardiography studies performed at lower volume centres (≤800 stress echo studies per annum, *n* = 8) pre-COVID, during $W_1$ and during $W_2$. $W_1$ – first wave, $W_2$ – second wave.

from 4 ± 0 pre-COVID to 2.7 ± 0.4 during $W_1$ and 2.9 ± 0.3 during $W_2$.

Using data from sites that indicated their numbers of stress echo sessions during both $W_1$ and $W_2$, Fig. 3 shows an overall 55% reduction in the estimated number of studies performed per month compared to pre-COVID-19 rates (78 ± 65 per month pre-COVID-19 vs 35 ± 32 per month during $W_1$, $P < 0.05$). The estimated number of studies performed during $W_2$ increased to more than reported during $W_1$ (71 ± 79 per month during $W_2$ vs 35 ± 32 per month during $W_1$, $P < 0.05$). The estimated number of studies performed during $W_2$ was not significantly different to pre-COVID-19 rates (71 ± 79 per month during $W_2$ vs 78 ± 65 per month pre-COVID-19, $P > 0.05$).

Figure 3B shows that in higher volume sites, the estimated number of studies per week fell from 116 studies per week pre-COVID to 58 per week in $W_1$ (50% fall, $P < 0.05$). This recovered to 125 per week in $W_2$ (108% of pre-COVID levels). In smaller sites (Fig. 3B), studies per week fell from 40 studies per week pre-COVID to 17 per week in $W_1$ (42% of pre-COVID levels, $P < 0.05$). This recovered to 47 per week in $W_2$ (118% of pre-COVID levels).

Figure 2B shows that of the 20 sites that reported use of exercise stressor pre-COVID-19, 14 sites (70%) had stopped their exercise stress echocardiography (ESE) service either due to ceasation of all stress echocardiography (four sites – 20%) or had replaced it with a dobutamine stress only service (ten sites – 50%) during $W_1$. The six sites (30%)

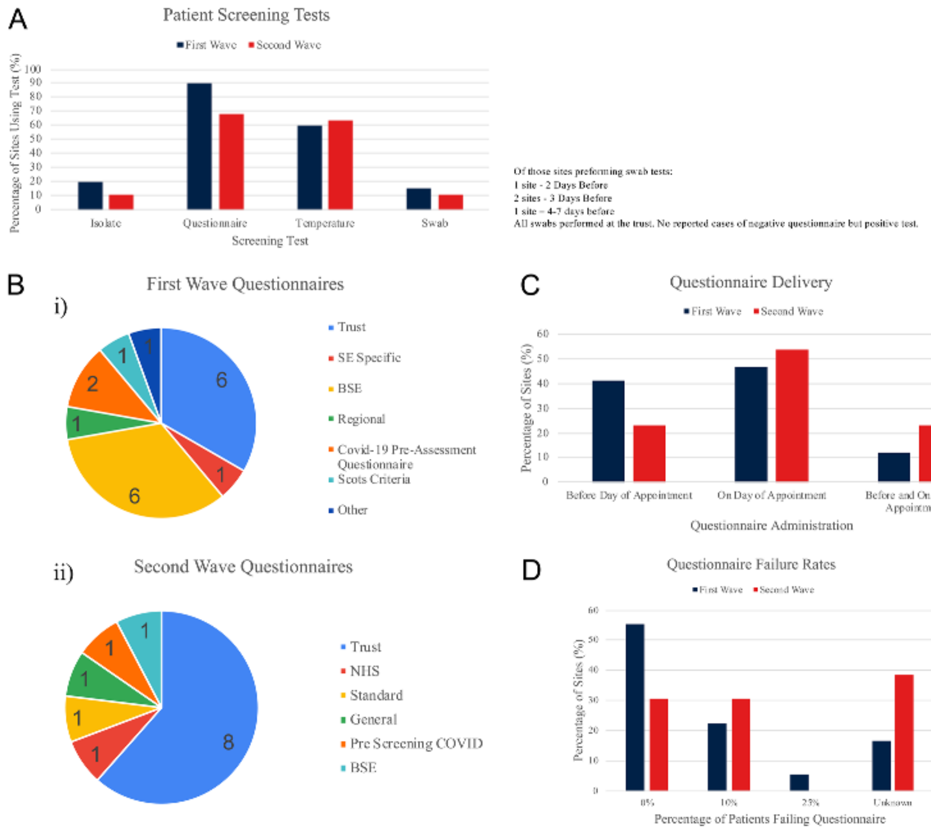

**Figure 4**

(A) The COVID-19 screening tests currently performed by the NHS sites performing stress echo during the first wave (*n* = 20) and second wave (*n* = 19). (B) Pie chart illustrating the different questionnaires used by the NHS sites during the first wave (i – *n* = 18) and second wave (ii – *n* = 13). (C) The point in the patient's pathway during which the questionnaire is administered at sites during the first wave (*n* = 18) and second wave (*n* = 13). (D) Percentages of patients failing the COVID-19 screening questionnaire during the first wave (*n* = 18) and second wave (*n* = 13).

continuing their exercise stress service during $W_1$ reported reduced the number of patients and a requirement to wear Level 2 PPE (fluid repellent disposable gown, respirator mask, gloves, eye protection).

Of the 16 sites who responded during $W_2$ and performed exercise stress pre-COVID-19, three sites (19%) had stopped their ESE service during $W_2$. Of the 13 sites still performing ESE during $W_2$, six sites (46%) reported that their ESE service was operating at a normal pre-COVID-19 rate.

## Impact on patients and personal protective equipment

Figure 4A illustrates the screening procedures used at the sites across the two waves. Sixty per cent of sites performed temperature checks during $W_1$ and 63% did so during $W_2$. During $W_1$, two sites performed COVID-19 swab tests between 2 and 3 days prior to the appointment, with one site performing swab test after the stress echo. The two sites performing swabs in $W_2$ did so 72 h and 4–7 days pre-appointment, respectively. Twenty per cent of sites asked patients to self-isolate during $W_1$, with two sites requiring 7 days and two 14 days. During $W_2$, two sites (11%) required patients to self-isolate for 3 days (at one site) or 4 days (at the other) after their swab test, prior to their appointment.

Figures 4B, C and D provide more detail on the use of screening questionnaires at sites. Eighteen sites (90%) asked patients to complete a health questionnaire during $W_1$, while 13 (68%) sites used questionnaires during $W_2$. Seven different questionnaires were used by sites during $W_1$, with most sites using either trust-derived (six sites – 33%) and BSE (six sites – 33%) questionnaires. Six different questionnaires were used during $W_2$, with the majority of sites using trust-derived questionnaires (eight sites – 62%). The questionnaires were administered pre-appointment (41% in $W_1$, 23% in $W_2$), at the appointment (47% in $W_1$, 54% in $W_2$) and both pre-appointment and on the day (12% in $W_1$, 23% in $W_2$). Twenty-five per cent https://erp.bioscientifica.com
https://doi.org/10.1530/ERP-20-0043
© 2020 The authors
Published by Bioscientifica Ltd

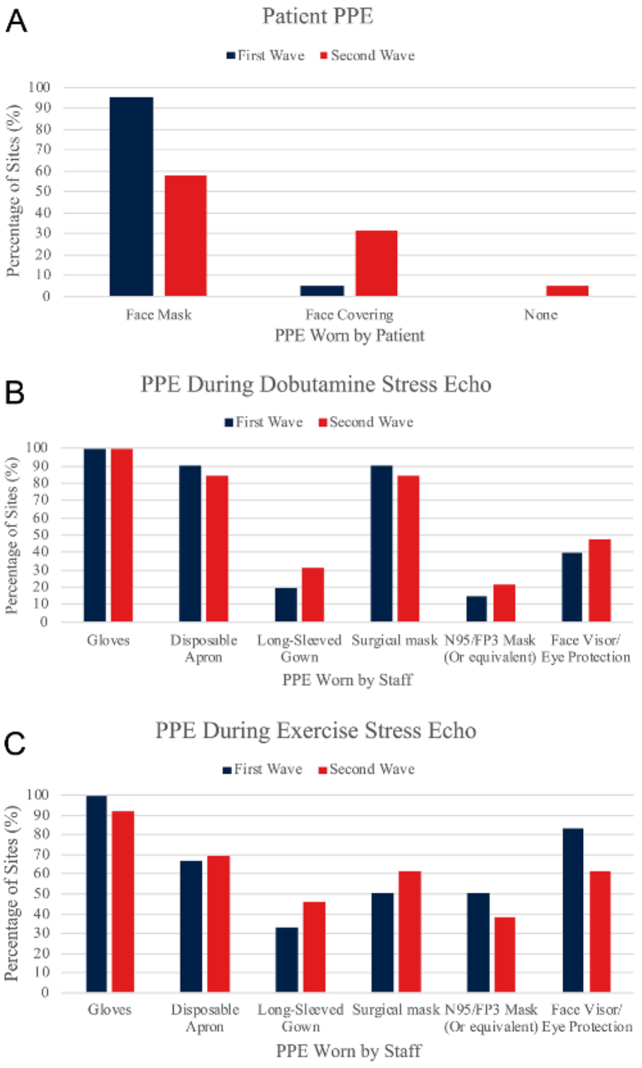

**Figure 5**
(A) The PPE required for patients to wear during stress echo at sites during the first wave (*n* = 20) and second wave (*n* = 19). (B) The percentage of sites requiring staff to wear each item of personal protective equipment during dobutamine stress echo in the first wave (*n* = 20) and second wave (*n* = 19). (C) The percentage of the sites performing exercise stress echocardiography requiring staff to wear each item of personal protective equipment during the procedure in the first wave (*n* = 6) and second wave (*n* = 13).

of patients had not proceeded to stress echo based on the questionnaire at one site during the first wave. No sites reported this incidence rate during the second wave. Ten per cent of patients did not proceed at 22% of sites in $W_1$ and 31% of sites during $W_2$. The remainder reported the questionnaire had not identified any patients. No cases of patients passing the questionnaire with a positive swab test were reported across both waves at all sites.

All sites, with the exception of one site during $W_2$, performing stress echocardiography reported that every patient is required to wear a face mask/covering for the procedure (Fig. 5A). For dobutamine stress echocardiography, surgical masks were worn by staff in 90% of sites during $W_1$ and 84% during $W_2$. Fifty per cent and 62% of sites reported use of surgical masks for exercise echocardiography during $W_1$ and $W_2$, respectively. The other sites required FFP3/N95 masks.

## Staff and ongoing impact

During $W_1$, 42% of sites were reviewing their stress echo practice weekly, falling to 5% of sites reviewing practice weekly during $W_2$ (Fig. 6A). During $W_1$, the majority of sites (54.2%) reported that none of their staff were unable to perform stress echocardiography due to COVID-19. This number fell to 37% during $W_2$, with 11% of sites reporting 25–50% of their staff were affected (compared to 0% during $W_1$) (Fig. 7A). The effects of COVID-19 on resting echocardiography are also reported in Fig. 7B.

## Discussion

This study shows stress echocardiography practice had restarted within a few months of the peak infection rate in

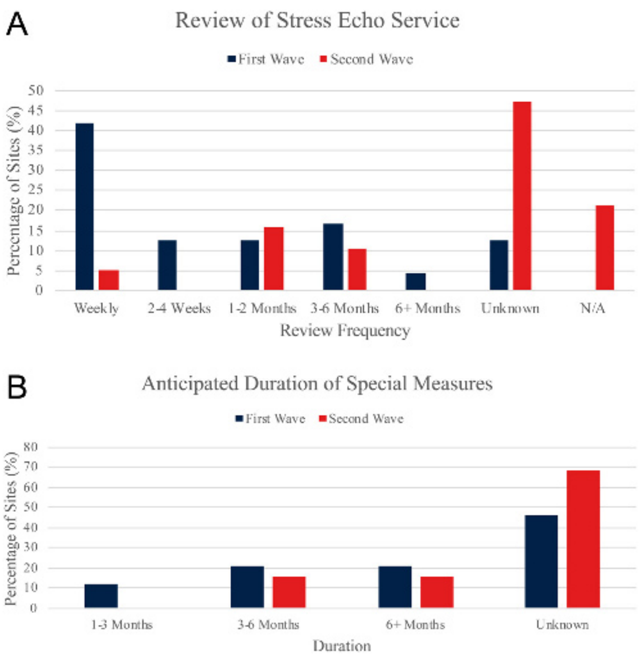

**Figure 6**
(A) The frequency with which the NHS sites reviewed the changes made to stress echocardiography practice at their site during the first wave (*n* = 24) and second wave (*n* = 19). (B) Duration of which the NHS sites anticipated the special measures put in place at their site, due to the COVID-19 pandemic, to continue for as collected during the first wave (*n* = 24) and second wave (*n* = 19).

C **First Wave**

| **DSE** | None | <10% | 10-25% |
|---|---|---|---|
| Surgical Mask | 10 | 3 | 3 |
| Level 2 Mask | 3 | 0 | 0 |
| **ESE** | None | <10% | 10-25% |
| Surgical Mask | 2 | N/A | 0 |
| Level 2 Mask | 3 | N/A | 1 |

D **Second Wave**

| **DSE** | None | <10% | 10-25% | 25-50% |
|---|---|---|---|---|
| Surgical Mask | 7 | 3 | 3 | 1 |
| Level 2 Mask | 1 | 2 | 0 | 1 |
| **ESE** | None | <10% | 10-25% | 25-50% |
| Surgical Mask | 2 | 2 | 1 | 1 |
| Level 2 Mask | 2 | 2 | 1 | 0 |

**Figure 7**
(A) The number of staff who were unable to perform stress echocardiography at the NHS sites due to COVID-19 during the first wave (*n* = 24) and second wave (*n* = 19). (B) The number of staff who were unable to perform resting echocardiography at theNHS sites due to COVID-19 during the first wave (*n* = 24) and second wave (*n* = 19). (C) The association between the level of PPE used and staff absence rates for the sites performing dobutamine and exercise stress echocardiography during the first wave. (D) The association between the level of PPE used and staff absence rates for the sites performing dobutamine and exercise stress echocardiography during the second wave.

the majority of hospitals during the first wave of COVID-19 hospital admissions, albeit at a reduced rate. During the second wave of COVID-19, stress echocardiography practice has been able to operate at a level not significantly different from pre-COVID-19 rates. The impact of the first wave and subsequent recovery in $W_2$ was not different between high and low volume centres.

The reduction in capacity during $W_1$ is likely to have generated a significant backlog of patients and although our findings suggest a return to similar levels of activity in $W_2$, this will not have been sufficient to clear this backlog. The reduction in capacity during $W_1$ may have been mitigated by use of clinical triage to identify potentially inappropriate requests or suggest transfer to alternative imaging tests. However, the return to normal activity in $W_2$ would suggest that any rationing or redistribution of care was only required for a short period. This is supported by our observation using data from the EVAREST study that rates of positive stress studies were very similar during November 2020 to January 2021 (14%) as before the onset of the COVID pandemic (18%).

The major change in practice during $W_1$ was a shift from exercise to dobutamine being used as the stressor. By $W_2$ most exercise stress services have restarted. The number of patients seen per session was reduced during both waves, likely to allow for aerosol dispersion and cleaning. The slight increase in the number of stress echo clinics per week during the second wave likely reflects a compensation to maintain patient numbers when there is an average reduction in number of patients per list. However, a significant heterogeneity in use of PPE and patient preparation prior to stress echocardiography is evident in both the waves.

The major variation between sites was the selection of screening tests for risk of COVID-19 in individual patients. Current BSE guidance recommends varying degrees of screening, from a COVID symptom questionnaire for DSE and TOE patients up to asking patinets to self-isolate for 2 weeks followed by a negative swab test 72/48 h before their exercise stress echo. The guidance suggests that the intensity of screening should be adjusted according to the current local prevalence of COVID positive cases (12).

Variation also exists in the use of PPE with only 38% of sites still performing exercise stress during $W_2$ using Level 2 personal protective equipment. Departmental policies on use of PPE did not appear to have any significant association with staff absence rate. There is a paucity of data with regard to the aerosol generating potential of exercise stress echocardiography. British Society of Echocardiography guidance states that the consensus opinion amongst stress echo experts in the United Kingdom is that exercise stress echocardiography may be considered an aerosol generating procedure (12). There is, therefore, a need for more investigation into whether exercise stress echocardiography has an increased risk of infection and, until evidence to the contrary, current BSE guidance has remained unchanged. It is possible that the wider use of faster testing and even vaccination passports, https://erp.bioscientifica.com
https://doi.org/10.1530/ERP-20-0043
© 2020 The authors
Published by Bioscientifica Ltd as the vaccination programme continues to expand, could be integrated into stress echocardiography practice to reduce transmission.

It is important to note that the results for $W_1$ presented in this paper were collected in July, several weeks after the first peak of the COVID-19 pandemic in the United Kingdom, when stress echocardiography practice may have been more adversely affected by staff redeployment and infection rates. Additionally, the numbers of studies performed per month during the two waves are estimated, based on the reported number of sessions per week. Hard data on the number of studies would provide a more robust evaluation of the effect of the pandemic on the number of studies performed.

In summary, while the number of studies performed did fall during the first wave of infections, there has been no long lasting impact on ability to deliver stress echocardiography within the NHS during the COVID-19 pandemic. During the second wave, most services were operating at normal rates and services had fully adapted to take account of requirements to reduce the risk of exposure through use of alternative stressors and PPE. Significant heterogeneity in screening tests and personal protective equipment used may require standardized national guidance to ensure consistency but local flexibility of service design may explain the apparent resilience of the cardiology centres to deliver stress echocardiography during a pandemic.

### Supplementary materials

This is linked to the online version of the paper at https://doi.org/10.1530/ERP-20-0043.

### Declaration of interest

P L is a shareholder and non-executive director of Ultromics, which develops AI echocardiography software, has previously consulted for Intelligent Ultrasound and has held research grants from the ultrasound contrast company Lantheus Medical Imaging. P L is an inventor on patents in the field of echocardiography.

### Funding

NIHR HEE Healthcare Science Research Fellowship (NIHR-HCS-P13-04-001); Cardiovascular Clinical Research Facility, University of Oxford; Ultromics Ltd.; Lantheus Medical Imaging Inc.; NIHR Oxford Biomedical Research Centre, University of Oxford.

### Author contribution statement

All authors were responsible for the design of the EVAREST COVID-19 impact survey. A M, C D and W W disseminated the survey and collated results. C D performed data analysis. C D, D A and P L drafted the manuscript. The final version was reviewed by all authors and investigators.
